# 2,4-Dichloro­benzyl 2-meth­oxy­benzoate

**DOI:** 10.1107/S1600536813006156

**Published:** 2013-03-09

**Authors:** Arun M. Isloor, B. Garudachari, Thomas Gerber, Eric Hosten, Richard Betz

**Affiliations:** a National Institute of Technology-Karnataka, Department of Chemistry, Surathkal, Mangalore 575 025, India; b Nelson Mandela Metropolitan University, Summerstrand Campus, Department of Chemistry, University Way, Summerstrand, PO Box 77000, Port Elizabeth, 6031, South Africa

## Abstract

In the title compound, C_15_H_12_Cl_2_O_3_, the aromatic rings make a dihedral angle of 10.78 (4)°. In the molecule, there is a short C—H⋯O contact. In the crystal, C—H⋯O contacts connect the mol­ecules into *C*(7)*C*(8) chains along the *b* axis. The shortest inter­centroid distance between two benzoic acid aromatic systems is 3.7416 (8) Å.

## Related literature

For pharmacological properties of phenyl benzoates, see: Oxford *et al.* (2005 ▶); Ostergaard (1994 ▶). For graph-set analysis of hydrogen bonds, see: Bernstein *et al.* (1995 ▶); Etter *et al.* (1990 ▶).
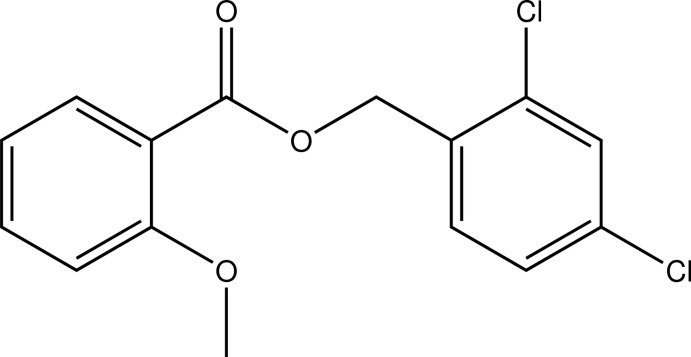


## Experimental

### 

#### Crystal data

C_15_H_12_Cl_2_O_3_
*M* *_r_* = 311.15
Monoclinic, 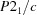

*a* = 12.1816 (5) Å
*b* = 15.2481 (6) Å
*c* = 7.4207 (4) Å
β = 99.299 (2)°
*V* = 1360.25 (11) Å^3^
*Z* = 4
Mo *K*α radiation
μ = 0.48 mm^−1^
*T* = 200 K
0.38 × 0.37 × 0.15 mm

#### Data collection

Bruker APEXII CCD diffractometer
Absorption correction: multi-scan (*SADABS*; Bruker, 2008 ▶) *T*_min_ = 0.847, *T*_max_ = 0.982
12933 measured reflections
3379 independent reflections
2868 reflections with *I* > 2σ(*I*)
*R*_int_ = 0.015

#### Refinement

*R*[*F* ^2^ > 2σ(*F* ^2^)] = 0.029
*wR*(*F* ^2^) = 0.080
*S* = 1.03
3379 reflections
182 parameters
H-atom parameters constrained
Δρ_max_ = 0.26 e Å^−3^
Δρ_min_ = −0.23 e Å^−3^

### 

Data collection: *APEX2* (Bruker, 2010 ▶); cell refinement: *SAINT* (Bruker, 2010 ▶); data reduction: *SAINT*; program(s) used to solve structure: *SHELXS97* (Sheldrick, 2008 ▶); program(s) used to refine structure: *SHELXL97* (Sheldrick, 2008 ▶); molecular graphics: *ORTEP-3 for Windows* (Farrugia, 2012 ▶) and *Mercury* (Macrae *et al.*, 2008 ▶); software used to prepare material for publication: *SHELXL97* and *PLATON* (Spek, 2009 ▶).

## Supplementary Material

Crystal structure: contains datablock(s) I, global. DOI: 10.1107/S1600536813006156/ng5318sup1.cif

Structure factors: contains datablock(s) I. DOI: 10.1107/S1600536813006156/ng5318Isup2.hkl

Supplementary material file. DOI: 10.1107/S1600536813006156/ng5318Isup3.cdx

Supplementary material file. DOI: 10.1107/S1600536813006156/ng5318Isup4.cml

Additional supplementary materials:  crystallographic information; 3D view; checkCIF report

## Figures and Tables

**Table 1:** Hydrogen-bond geometry (Å, °)

*D*—H⋯*A*	*D*—H	H⋯*A*	*D*⋯*A*	*D*—H⋯*A*
C3—H3*A*⋯O2^i^	0.98	2.59	3.5433 (18)	165
C15—H15⋯O2^i^	0.95	2.51	3.3945 (15)	154
C16—H16⋯O3	0.95	2.48	3.3989 (15)	163

Symmetry code: (i) 
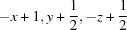
.

**Fig. 1.**
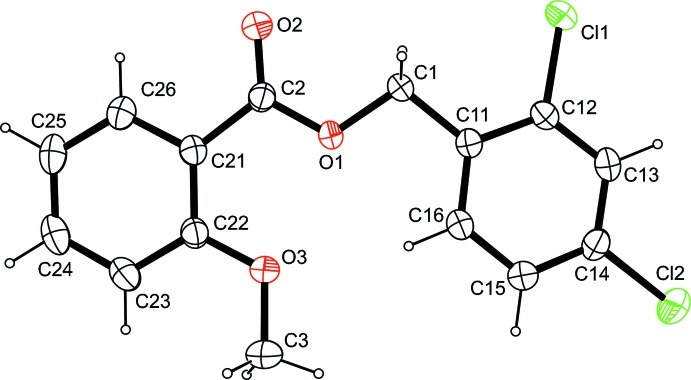
The molecular structure of the title compound, with atom labels and anisotropic displacement ellipsoids (drawn at 50% probability level).

**Fig. 2.**
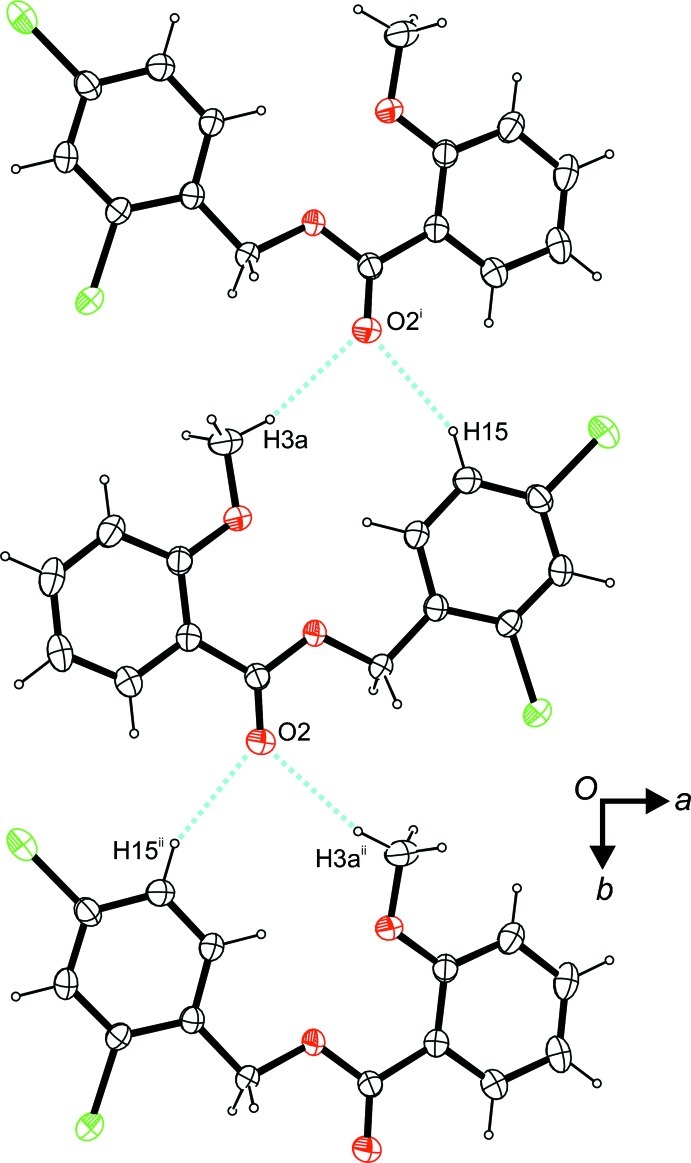
Intermolecular contacts, viewed along [0 0 1]. Symmetry operators: i -x + 1, y + 1/2, -z + 1/2; ii -x + 1, y - 1/2, -z + 1/2.

**Fig. 3.**
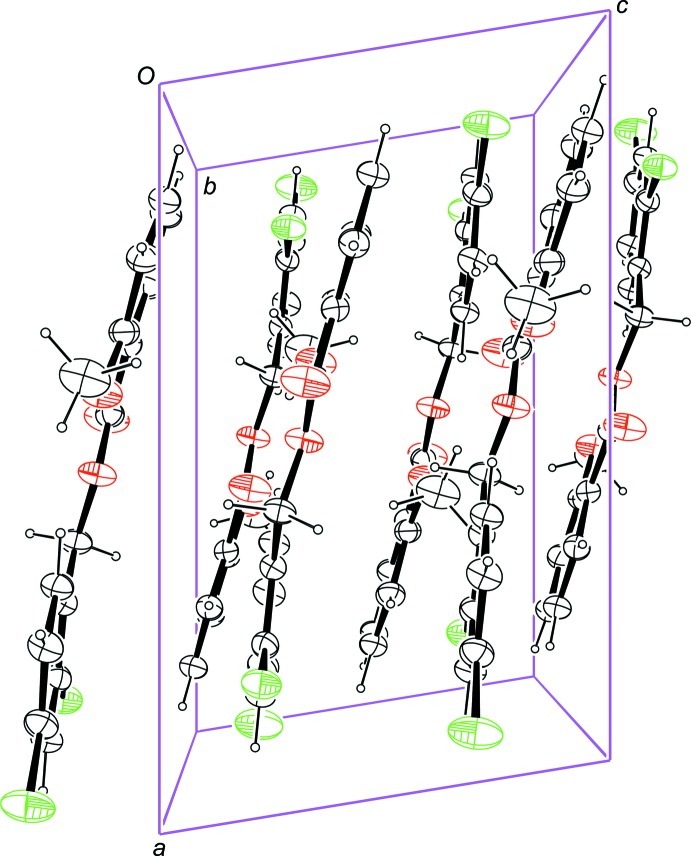
Molecular packing of the title compound, viewed along [0 1 0] (anisotropic displacement ellipsoids drawn at 50% probability level).

## supplementary crystallographic information

### Comment

For decades, phenyl benzoate derivatives have found ample application in the identification of organic acids. 2,4-Dichlorobenzyl derivatives show pharmacological activity such as acting as mild antiseptics and showing the ability to kill bacteria and viruses associated with mouth and throat infections (Oxford *et al.*, 2005; Ostergaard 1994). In continuation of our research focused on the crystal structures of medical compounds, the title compound was synthesized.

The molecule is essentially flat. The atom deviating most from the least-squares plane defined by all non-hydrogen atoms is the chlorine atom in *para* position to the benzyloxy moiety with a deviation of 0.237 (1) Å. The least-squares planes defined by the respective carbon atoms of the aromatic systems intersect at an angle of 10.78 (4) ° (Fig. 1).

In the crystal, intermolecular C–H···O contacts whose range falls by more than 0.1 Å below the sum of van-der-Waals radii are observed. These are established between one of the hydrogen atoms of the methoxy group as well as one of the two hydrogen atoms in *ortho* position to the chlorine atom in *para* position to the benzyloxy group and chelate the ketonic oxygen atom of the ester moiety. In total, the molecules are connected to chains along the crystallographic *b* axis. In terms of graph-set analysis (Etter *et al.*, 1990; Bernstein *et al.*, 1995), the descriptor for these contacts is *C*^1^_1_(7)*C*^1^_1_(8) on the unary level. Information about metrical parameters as well as the symmetry of those contacts has been summarized in Table 1. The shortest intercentroid distance between two aromatic systems was measured at 3.7416 (8) Å and is apparent between the benzoic acid moiety and its symmetry-generated equivalent (Fig. 2).

The packing of the title compound in the crystal structure is shown in Figure 3.

### Experimental

A mixture of 1-(bromomethyl)-2,4-dichlorobenzene (0.1 g, 0.0004 mol), potassium carbonate (0.062 g, 0.00045 mol) and 2-methoxybenzoic acid (0.068 g, 0.00045 mol) in dimethylformamide (5 ml) was stirred at 60 °C for 2 h. After completion of the reaction, the reaction mixture was poured into ice-cold water. The solid product obtained was filtered, washed with water and recrystallized from ethanol (yield: 0.120 g, 93.0%).

### Refinement

Carbon-bound H atoms were placed in calculated positions (C–H 0.95 Å for aromatic carbon atoms and C–H 0.99 Å for methylene groups) and were included in the refinement in the riding model approximation, with *U*(H) set to 1.2*U*_eq_(C). The H atoms of the methyl groups were allowed to rotate with a fixed angle around the C–C bond to best fit the experimental electron density with *U*(H) set to 1.5*U*_eq_(C).

### Crystal data

**Table:**

C_15_H_12_Cl_2_O_3_	*F*(000) = 640
*M**_r_* = 311.15	*D*_x_ = 1.519 Mg m^−^^3^
Monoclinic, *P*2_1_/*c*	Melting point = 376–374 K
Hall symbol: -P 2ybc	Mo *K*α radiation, λ = 0.71073 Å
*a* = 12.1816 (5) Å	Cell parameters from 6565 reflections
*b* = 15.2481 (6) Å	θ = 2.7–28.3°
*c* = 7.4207 (4) Å	µ = 0.48 mm^−^^1^
β = 99.299 (2)°	*T* = 200 K
*V* = 1360.25 (11) Å^3^	Block, colourless
*Z* = 4	0.38 × 0.37 × 0.15 mm

### Data collection

**Table:**

Bruker APEXII CCD diffractometer	3379 independent reflections
Radiation source: fine-focus sealed tube	2868 reflections with *I* > 2σ(*I*)
Graphite monochromator	*R*_int_ = 0.015
φ and ω scans	θ_max_ = 28.4°, θ_min_ = 2.2°
Absorption correction: multi-scan (*SADABS*; Bruker, 2008)	*h* = −16→16
*T*_min_ = 0.847, *T*_max_ = 0.982	*k* = −20→20
12933 measured reflections	*l* = −9→9

### Refinement

**Table:**

Refinement on *F*^2^	Primary atom site location: structure-invariant direct methods
Least-squares matrix: full	Secondary atom site location: difference Fourier map
*R*[*F*^2^ > 2σ(*F*^2^)] = 0.029	Hydrogen site location: inferred from neighbouring sites
*wR*(*F*^2^) = 0.080	H-atom parameters constrained
*S* = 1.03	*w* = 1/[σ^2^(*F*_o_^2^) + (0.0407*P*)^2^ + 0.3593*P*] where *P* = (*F*_o_^2^ + 2*F*_c_^2^)/3
3379 reflections	(Δ/σ)_max_ = 0.001
182 parameters	Δρ_max_ = 0.26 e Å^−^^3^
0 restraints	Δρ_min_ = −0.23 e Å^−^^3^

### Fractional atomic coordinates and isotropic or equivalent isotropic displacement parameters (Å^2^)

**Table:**

	*x*	*y*	*z*	*U*_iso_*/*U*_eq_	
Cl1	0.83729 (2)	0.13621 (2)	0.22173 (5)	0.03453 (10)	
Cl2	0.93726 (3)	0.47791 (2)	0.20393 (6)	0.05086 (12)	
O1	0.50796 (7)	0.23368 (5)	0.32072 (13)	0.0302 (2)	
O2	0.42680 (8)	0.10301 (6)	0.32410 (15)	0.0417 (2)	
O3	0.39479 (7)	0.37220 (5)	0.34997 (14)	0.0354 (2)	
C1	0.60253 (9)	0.19251 (7)	0.26253 (17)	0.0260 (2)	
H1A	0.5802	0.1629	0.1435	0.031*	
H1B	0.6354	0.1483	0.3532	0.031*	
C2	0.42351 (9)	0.18141 (7)	0.34359 (16)	0.0250 (2)	
C3	0.37406 (13)	0.46402 (9)	0.3289 (2)	0.0465 (4)	
H3A	0.4363	0.4920	0.2823	0.070*	
H3B	0.3664	0.4896	0.4473	0.070*	
H3C	0.3053	0.4735	0.2425	0.070*	
C11	0.68490 (9)	0.26426 (7)	0.24577 (15)	0.0234 (2)	
C12	0.79427 (9)	0.24488 (7)	0.22730 (16)	0.0244 (2)	
C13	0.87222 (10)	0.30935 (8)	0.21331 (17)	0.0291 (3)	
H13	0.9464	0.2945	0.2012	0.035*	
C14	0.83948 (10)	0.39622 (8)	0.21735 (17)	0.0303 (3)	
C15	0.73151 (10)	0.41879 (8)	0.23178 (18)	0.0306 (3)	
H15	0.7097	0.4786	0.2319	0.037*	
C16	0.65539 (10)	0.35254 (8)	0.24611 (17)	0.0274 (2)	
H16	0.5810	0.3678	0.2564	0.033*	
C21	0.32638 (9)	0.22915 (7)	0.39497 (16)	0.0249 (2)	
C22	0.31284 (9)	0.32091 (8)	0.39762 (16)	0.0268 (2)	
C23	0.21596 (10)	0.35594 (9)	0.44706 (18)	0.0327 (3)	
H23	0.2066	0.4177	0.4505	0.039*	
C24	0.13359 (10)	0.30135 (10)	0.49094 (18)	0.0355 (3)	
H24	0.0686	0.3260	0.5261	0.043*	
C25	0.14469 (10)	0.21127 (10)	0.48428 (18)	0.0351 (3)	
H25	0.0870	0.1740	0.5113	0.042*	
C26	0.24091 (10)	0.17634 (8)	0.43777 (17)	0.0296 (3)	
H26	0.2491	0.1144	0.4349	0.036*	

### Atomic displacement parameters (Å^2^)

**Table:**

	*U*^11^	*U*^22^	*U*^33^	*U*^12^	*U*^13^	*U*^23^
Cl1	0.02915 (16)	0.02768 (16)	0.0483 (2)	0.00585 (11)	0.01086 (13)	−0.00228 (12)
Cl2	0.03554 (19)	0.03606 (19)	0.0846 (3)	−0.00991 (14)	0.02065 (18)	0.00427 (17)
O1	0.0219 (4)	0.0242 (4)	0.0472 (5)	−0.0005 (3)	0.0138 (4)	−0.0033 (3)
O2	0.0334 (5)	0.0234 (4)	0.0723 (7)	−0.0010 (4)	0.0204 (5)	−0.0008 (4)
O3	0.0301 (4)	0.0218 (4)	0.0571 (6)	0.0015 (3)	0.0155 (4)	0.0014 (4)
C1	0.0220 (5)	0.0230 (5)	0.0345 (6)	0.0021 (4)	0.0090 (4)	−0.0009 (5)
C2	0.0220 (5)	0.0251 (5)	0.0280 (6)	−0.0011 (4)	0.0040 (4)	0.0020 (4)
C3	0.0460 (8)	0.0234 (6)	0.0740 (11)	0.0030 (6)	0.0218 (8)	0.0014 (6)
C11	0.0219 (5)	0.0261 (5)	0.0227 (5)	0.0005 (4)	0.0048 (4)	0.0003 (4)
C12	0.0244 (5)	0.0259 (5)	0.0235 (5)	0.0033 (4)	0.0052 (4)	−0.0005 (4)
C13	0.0218 (5)	0.0350 (6)	0.0318 (6)	0.0008 (5)	0.0086 (4)	0.0001 (5)
C14	0.0282 (6)	0.0295 (6)	0.0347 (6)	−0.0058 (5)	0.0092 (5)	0.0012 (5)
C15	0.0311 (6)	0.0252 (6)	0.0372 (7)	0.0006 (5)	0.0102 (5)	0.0013 (5)
C16	0.0231 (5)	0.0276 (6)	0.0329 (6)	0.0023 (4)	0.0085 (5)	0.0009 (5)
C21	0.0220 (5)	0.0277 (6)	0.0250 (6)	0.0003 (4)	0.0036 (4)	−0.0007 (4)
C22	0.0224 (5)	0.0291 (6)	0.0290 (6)	0.0003 (4)	0.0044 (4)	−0.0006 (5)
C23	0.0274 (6)	0.0362 (7)	0.0346 (7)	0.0064 (5)	0.0053 (5)	−0.0045 (5)
C24	0.0238 (6)	0.0517 (8)	0.0318 (6)	0.0049 (5)	0.0068 (5)	−0.0059 (6)
C25	0.0245 (6)	0.0493 (8)	0.0328 (7)	−0.0062 (5)	0.0089 (5)	−0.0022 (6)
C26	0.0271 (6)	0.0330 (6)	0.0293 (6)	−0.0041 (5)	0.0063 (5)	−0.0003 (5)

### Geometric parameters (Å, º)

**Table:**

Cl1—C12	1.7403 (12)	C13—C14	1.3849 (17)
Cl2—C14	1.7374 (12)	C13—H13	0.9500
O1—C2	1.3337 (13)	C14—C15	1.3804 (17)
O1—C1	1.4383 (13)	C15—C16	1.3869 (17)
O2—C2	1.2057 (15)	C15—H15	0.9500
O3—C22	1.3592 (14)	C16—H16	0.9500
O3—C3	1.4267 (15)	C21—C26	1.3929 (16)
C1—C11	1.5033 (15)	C21—C22	1.4093 (16)
C1—H1A	0.9900	C22—C23	1.3973 (16)
C1—H1B	0.9900	C23—C24	1.3826 (19)
C2—C21	1.4902 (16)	C23—H23	0.9500
C3—H3A	0.9800	C24—C25	1.382 (2)
C3—H3B	0.9800	C24—H24	0.9500
C3—H3C	0.9800	C25—C26	1.3811 (17)
C11—C12	1.3929 (16)	C25—H25	0.9500
C11—C16	1.3933 (16)	C26—H26	0.9500
C12—C13	1.3823 (17)		
			
C2—O1—C1	116.68 (9)	C15—C14—Cl2	119.76 (10)
C22—O3—C3	117.96 (10)	C13—C14—Cl2	118.81 (9)
O1—C1—C11	106.55 (9)	C14—C15—C16	118.79 (11)
O1—C1—H1A	110.4	C14—C15—H15	120.6
C11—C1—H1A	110.4	C16—C15—H15	120.6
O1—C1—H1B	110.4	C15—C16—C11	121.85 (11)
C11—C1—H1B	110.4	C15—C16—H16	119.1
H1A—C1—H1B	108.6	C11—C16—H16	119.1
O2—C2—O1	122.41 (11)	C26—C21—C22	118.51 (11)
O2—C2—C21	123.93 (11)	C26—C21—C2	115.44 (10)
O1—C2—C21	113.66 (10)	C22—C21—C2	126.02 (10)
O3—C3—H3A	109.5	O3—C22—C23	122.36 (11)
O3—C3—H3B	109.5	O3—C22—C21	118.37 (10)
H3A—C3—H3B	109.5	C23—C22—C21	119.27 (11)
O3—C3—H3C	109.5	C24—C23—C22	120.50 (12)
H3A—C3—H3C	109.5	C24—C23—H23	119.8
H3B—C3—H3C	109.5	C22—C23—H23	119.8
C12—C11—C16	117.15 (11)	C25—C24—C23	120.74 (12)
C12—C11—C1	121.04 (10)	C25—C24—H24	119.6
C16—C11—C1	121.81 (10)	C23—C24—H24	119.6
C13—C12—C11	122.41 (11)	C26—C25—C24	118.96 (12)
C13—C12—Cl1	117.53 (9)	C26—C25—H25	120.5
C11—C12—Cl1	120.05 (9)	C24—C25—H25	120.5
C12—C13—C14	118.35 (11)	C25—C26—C21	121.99 (12)
C12—C13—H13	120.8	C25—C26—H26	119.0
C14—C13—H13	120.8	C21—C26—H26	119.0
C15—C14—C13	121.43 (11)		
			
C2—O1—C1—C11	179.67 (10)	O2—C2—C21—C26	6.06 (18)
C1—O1—C2—O2	2.90 (17)	O1—C2—C21—C26	−173.90 (10)
C1—O1—C2—C21	−177.14 (10)	O2—C2—C21—C22	−171.70 (12)
O1—C1—C11—C12	167.09 (10)	O1—C2—C21—C22	8.34 (16)
O1—C1—C11—C16	−13.58 (15)	C3—O3—C22—C23	−7.76 (18)
C16—C11—C12—C13	1.25 (18)	C3—O3—C22—C21	171.63 (12)
C1—C11—C12—C13	−179.38 (11)	C26—C21—C22—O3	−177.87 (11)
C16—C11—C12—Cl1	−178.92 (9)	C2—C21—C22—O3	−0.17 (18)
C1—C11—C12—Cl1	0.44 (16)	C26—C21—C22—C23	1.53 (17)
C11—C12—C13—C14	−0.22 (18)	C2—C21—C22—C23	179.23 (11)
Cl1—C12—C13—C14	179.96 (9)	O3—C22—C23—C24	178.65 (12)
C12—C13—C14—C15	−1.10 (19)	C21—C22—C23—C24	−0.73 (18)
C12—C13—C14—Cl2	178.88 (9)	C22—C23—C24—C25	−0.9 (2)
C13—C14—C15—C16	1.3 (2)	C23—C24—C25—C26	1.7 (2)
Cl2—C14—C15—C16	−178.68 (10)	C24—C25—C26—C21	−0.90 (19)
C14—C15—C16—C11	−0.19 (19)	C22—C21—C26—C25	−0.73 (18)
C12—C11—C16—C15	−1.04 (18)	C2—C21—C26—C25	−178.67 (11)
C1—C11—C16—C15	179.60 (11)		

### Hydrogen-bond geometry (Å, º)

**Table:**

*D*—H···*A*	*D*—H	H···*A*	*D*···*A*	*D*—H···*A*
C3—H3*A*···O2^i^	0.98	2.59	3.5433 (18)	165
C15—H15···O2^i^	0.95	2.51	3.3945 (15)	154
C16—H16···O3	0.95	2.48	3.3989 (15)	163

Symmetry code: (i) −*x*+1, *y*+1/2, −*z*+1/2.
